# Strain Measurement Based on Speeded-Up Robust Feature Algorithm Applied to Microimages from a Smartphone-Based Microscope

**DOI:** 10.3390/s20102805

**Published:** 2020-05-15

**Authors:** Botao Xie, Jinke Li, Xuefeng Zhao

**Affiliations:** 1 School of Civil Engineering, Dalian University of Technology, Dalian 116024, China; botaoxie@mail.dlut.edu.cn (B.X.); jinkeli@mail.dlut.edu.cn (J.L.); 2 State Key Laboratory of Coastal and Offshore Engineering, Dalian University of Technology, Dalian 116024, China

**Keywords:** strain measurement, smartphone, microimages, speeded-up robust feature (SURF), M-estimator sample consensus (MSAC)

## Abstract

The objective of this study is to evaluate and improve the accuracy and stability of a strain measurement method that uses the speeded-up robust feature (SURF) method to trace the displacement of feature points in microimages and obtain the strain in objects. The microimages were acquired using a smartphone with a portable microscope, which has a broad prospect of application. An experiment was performed using an unpacked optical fiber as the experimental carrier. The matching effect of the SURF method was analyzed in the microimage, and the M-estimator sample consensus (MSAC) algorithm was used to reject outliers generated by SURF. The results indicated that the accuracy of strain measurement using the proposed method is improved by modifying the feature point tracking method and measurement method. When compared with the fiber Bragg grating (FBG) data, the maximum standard error corresponded to 2.5 με, which satisfies the requirement of structural health monitoring (SHM) in practical engineering.

## 1. Introduction

Strain is an important parameter that reflects a structural state, which can be used to evaluate the mechanical properties, failure behavior, crack development, and residual stress of structural members and materials. It plays an important role in the field of structural health monitoring (SHM). In the past 20 years, several extant studies focused on and developed various methods for measuring strain. The strain measurement methods can be divided into contact measurement and non-contact measurement methods. Contact measurement is mainly based on traditional measurement methods including the strain gauge, fiber Bragg grating (FBG) sensor, and fiber optic sensor. The sensors display high measurement accuracy and mature technology and are applied to SHM of large structures such as bridges [1,2,3,4], tunnels [5], and wind turbines [6]. However, the application of the sensors requires a variety of expensive instruments. Additionally, the sensors entail complex preparation, the instrument circuit layout is tedious, and it requires personnel with significant professional knowledge to operate. The requirements limit their application scope, and it is difficult to extend the same to a large number of common structures.

The non-contact measurement technology mainly corresponds to optical measurement technology. It exhibits the advantages of non-contact, non-destructive structure, and full field measurement. Hence, it has attracted significant research attention for strain measurement. Optical measurement technology can be divided into an interference method and non-interference method. The interference method mainly includes holographic interferometry [7], high sensitivity Moiré [8,9], and speckle interferometry [10]. The non-interference method is mainly represented by the digital image correlation method [11,12]. When compared with interference methods, non-interference methods do not require a complex optical path and environment. Hence, they can be widely used. Therefore, the digital image correlation (DIC) method is widely studied by researchers, and its applicability is also verified in practice [13,14]. However, the measurement accuracy of DIC technology is affected by the measurement equipment, noise, and algorithm, and therefore its measurement accuracy is relatively low. With the improvements in computing power and continuous development of image processing technology, it is possible to use digital image correlation analysis combined with various high-resolution digital image acquisition equipment, such as an optical microscope [15], confocal laser scanning microscope [16], scanning electron microscope [17,18] and atomic force microscope, [19,20] to measure the deformation at the micro–nano scale to improve the measurement accuracy. Nevertheless, due to the price, volume, weight, and other factors of high-precision microimaging equipment, it is difficult to utilize them in practical projects.

The aforementioned limitations require the development of a cheap, high-precision, and universal strain sensor. Hence, the application of a smartphone, microscope, and image feature detection can potentially resolve this issue. Recently, the development and application of smartphones led to a novel direction to SHM [21,22]. Smartphones exhibit a high penetration rate. They possess their own sensing function, data collection function, and data transmission function such that they can obtain a large number of monitoring data, which is applied in several extant studies to SHM [23,24,25,26,27,28,29]. With increases in the pixel values of smartphone cameras, many studies focused on the visual monitoring of construction safety and damage detection based on the image processing method [30,31,32]. Simultaneously, a mobile phone microscope matched with the smartphone is increasingly portable and can take pictures at the micro-scale, thereby laying the foundation for strain measurement using the smartphones. Based on the same information, the study used smartphones, a portable microscope, and an image feature detection method to measure the average strain of a certain length of optical fiber to simulate the process of measuring the surface strain of an object. Furthermore, the accuracy and stability of the method were verified and improved via experiments.

## 2. Experimental Details

### 2.1. Principle of FBG

The test object of the study involved a standard optical fiber SMF-28 (Corning, NY, USA), and an FBG sensor (Guang You, Hangzhou, China) was connected in series. The optical fiber diameter is 245 ± 5 μm and has the same protective coating as FBG such that FBG could accurately measure the strain of it. FBG is the periodic refractive-index structure in an optical fiber core, where the light of a specific wavelength is reflected. It can be used as a strain sensor by measuring the change of the reflective wavelength of FBG. Its central wavelength can be expressed as:

(1) $$\lambda_{B}=2n\Lambda$$

where λ_B_ denotes the central wavelength of FBG and is in nm. Λ denotes the grating period and n denotes the effective refractive index of the core. The central wavelength of FBG used in the experiment was 1550 nm. When FBG is deformed by force, the grid spacing of FBG will change, which will cause the change of wavelength reflection. In terms of the change of demodulation wavelength, the deformation (strain) of FBG can be deduced. The measuring strain can be expressed as Formula (2) [33]:

(2) $$\Delta \varepsilon =\frac{\Delta \lambda_{B}}{\lambda_{B}(1-P_{e})}$$

where Δε denotes the longitudinal strain, Δλ_B_ denotes the central wavelength change value, and $P_{e}=-\frac{1}{n}\frac{dn}{d\varepsilon }$ is an effective photoelastic constant. In 1990, Moery et al. [33] measured this constant of 1550 nm FBG as 1.209 pm/με.

When FBG is subjected to temperature change, its central wavelength will also change, which will affect the strain measurement results. However, the experiments in this study were carried out in the laboratory, and the time span of each experiment was about 5 min, which was not enough to produce obvious temperature change. Therefore, the influence of temperature on the strain results measured by FBG was not considered in this study.

### 2.2. Setup of the Test

**Figure 1:**
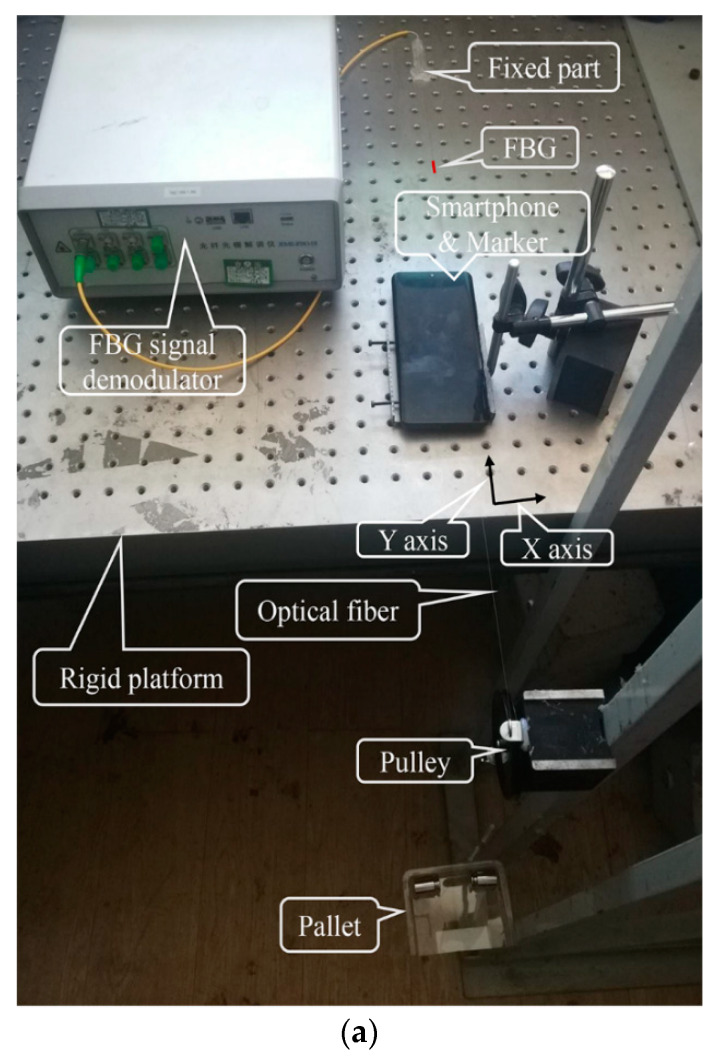

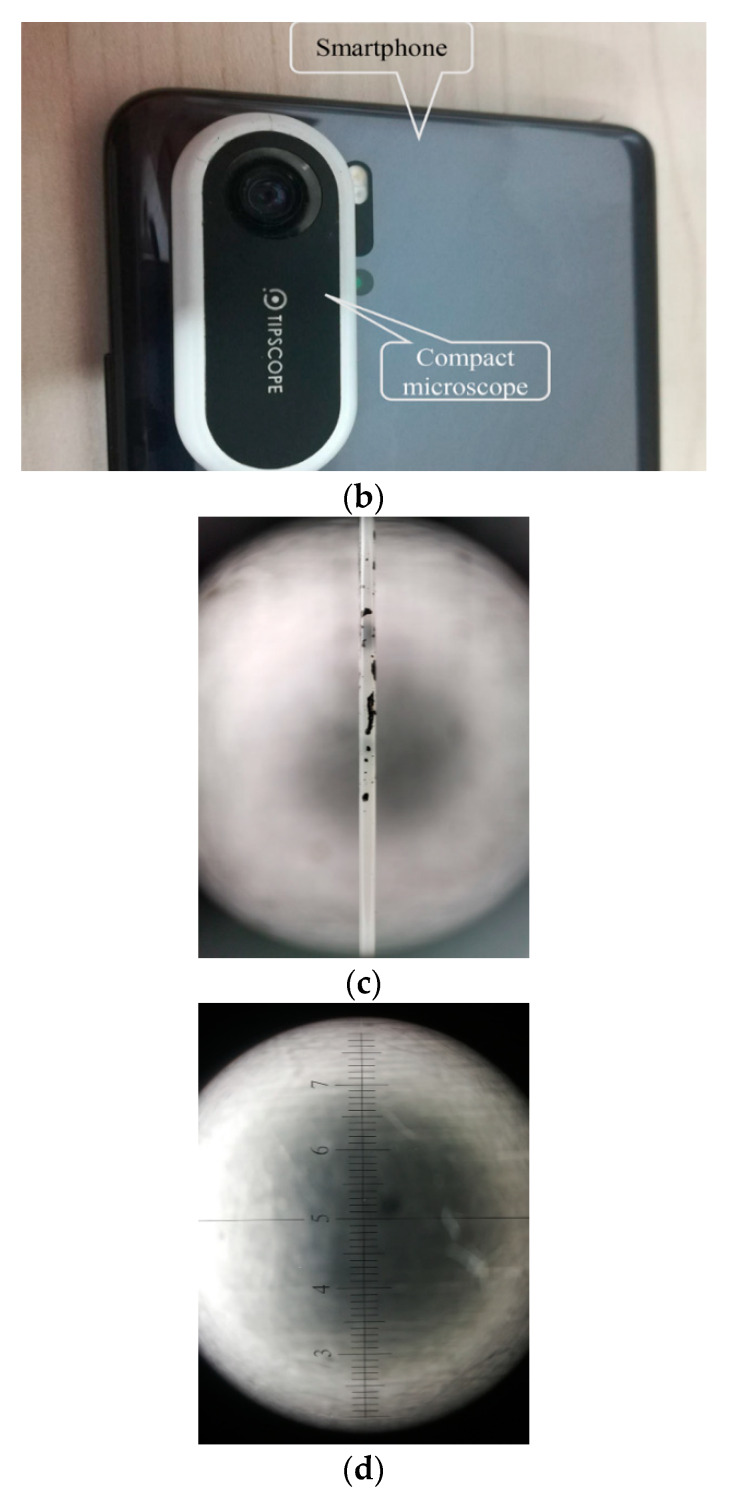
Schematic illustration of the (**a**) test, (**b**) compact microscope, (**c**) marker of optical fiber, and (**d**) micrometer imaged by the smartphone with a microscope.

**Figure 2:**
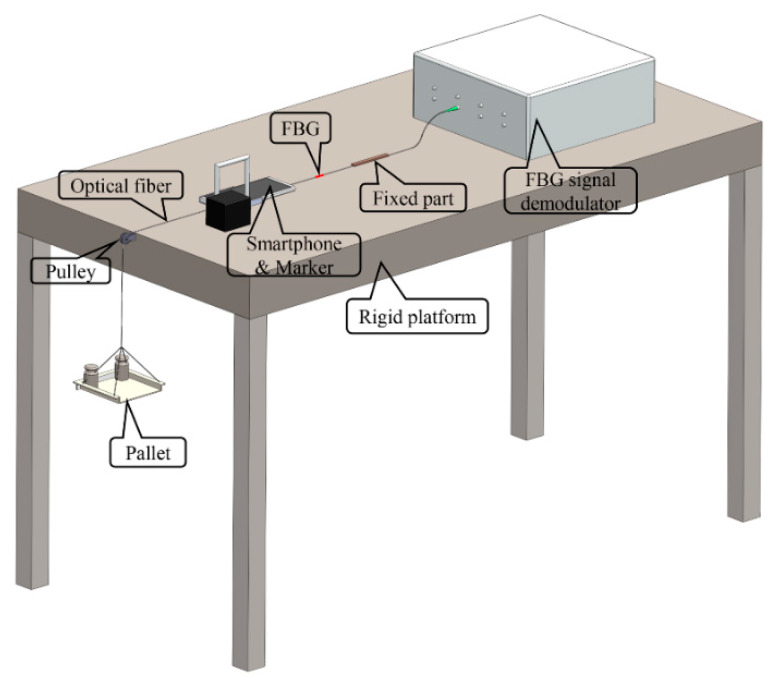
Setup of the test.

The test setup is shown in Figure 1a and Figure 2. As shown in the figures, the test equipment consists of an optical fiber, an FBG sensor, an FBG demodulator, a rigid platform, a pallet, pulley, weights, smartphones, and a compact microscope. A force was simultaneously applied to the optical fiber and FBG, and the material of the optical fiber corresponded to a uniform linear elastic material [34] such that the strain observed by the smartphone was identical to the strain measured by the FBG. The microscope is referred to as TIPSCOPE and can be conveniently attached on the camera of the smartphone, as shown in Figure 1b. The microscope exhibited a magnification of 20–400 times, and the weight was 3 g, the thickness was only 5 mm, and the focal length was 20 mm. When coupled with a smartphone, the diameter of the field of view in the microimage captured is about 5 mm. Meanwhile, the flashlight of the smartphone can be used as the light source of the microscope to ensure a clear image. A marker (feature points) for image detection is placed at a certain distance on the optical fiber from the fixed part, as shown in Figure 1c, which corresponded to the observation point of the smartphone attached with a microscope. In order to measure the actual size of each pixel in the image obtained by the smartphone, the pixel in the image was calibrated using a micrometer with an accuracy of 0.1 mm before the experiment started, as shown in Figure 1d.

The measuring distance, which is the distance between the fixed part and marker in the experiment, corresponded to 30 cm, and the weight used for loading corresponded to 1 g. Two smartphones with different pixel values were used in the study. Each smartphone can be set to two different shooting pixel values. The resolution of smartphones was in megapixels (MP), which corresponded to 8, 10, 16, and 40 MP. The resolution of one of the smartphones corresponded to 8 and 16 MP, and that of the other smartphone corresponded to 10 and 40 MP. The four pixel values essentially covered the resolution of the existing smartphones in the market. The actual moving displacement of the features was obtained by tracking the position of the features on the optical fiber in the image and combining the same with the pixel value for 1-mm distance in the micrometer. Using the actual distance L, the strain of the optical fiber under the tensile force was also obtained. In order to observe the stability of the proposed method, the experiment performed five repeatability experiments on each smartphone with different pixel values, and the results were compared with the FBG measurement data. Table 1 lists the test cases.

## 3. Measuring Principle

**Figure 3:**
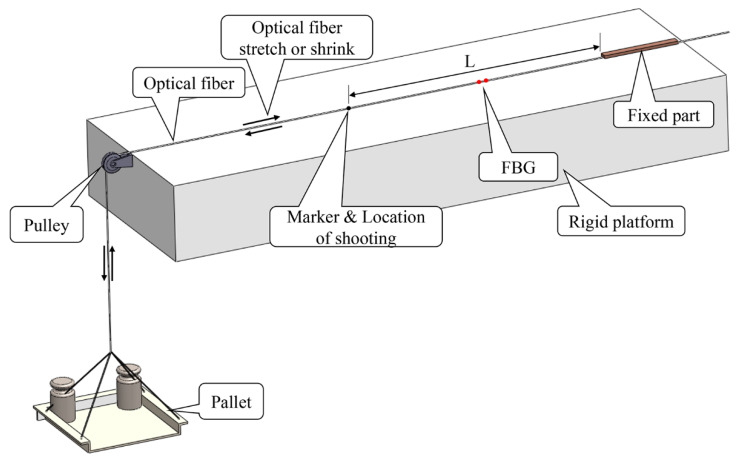
Partial diagram of the test.

The partial schematic diagram of the experiment is shown in Figure 3. As shown in the figure, the length from the marker on the optical fiber to the fixed part corresponds to L (which represents the length of the measured object) and corresponds to 30 cm in the experiment. We photograph and record the pictures of the marker before and after loading as the reference case and deformation case, respectively, by using the smartphones. By identifying and tracking the position of the marker in the image before and after loading, the displacement of the marker after deformation is calculated, and the unit corresponds to pixels. Based on the actual size of each pixel in the calibrated image, the actual displacement value of the marker is also calculated, and the unit corresponds to mm. The average strain of the measuring section is obtained by dividing the actual moving displacement by the actual length L of the measuring section. Because the diameter of microimages captured was about 5 mm and the measurement distance L was 30 cm, the limit of detection (LOD) of the strain measurement based on the proposed method in the experiment was about −8.3 × 10^3^~8.3 × 10^3^ με.

Table 2 shows the number of pixels in the image recorded by smartphones with different pixel values calibrated in micrometers and the actual strain represented by each pixel.

As shown in Table 2, as the pixel value of the smartphone decreases, the number of pixels per millimeter also decreases, and this in turn decreases the actual strain per pixel. However, based on the data in the table, the accuracy of the actual strain represented by each pixel of smartphones with different pixel values can essentially satisfy the engineering requirements, and the image detection algorithm used in the study operates at a sub-pixel level. Thus, the accuracy of the measurements increases, as demonstrated in the next sections. The aforementioned analysis indicates that the tracking effect of the marker in the image corresponds to a key factor that affects the accuracy and stability of the proposed method.

## 4. Speeded-Up Robust Feature Method

In the study, the SURF method was used to detect the displacement of feature points. Specifically, SURF is an image feature detection algorithm proposed by Bay et al. [35]. The algorithm is an improvement of the scale-invariant feature transform (SIFT) [36,37]. It includes the advantages of SIFT to maintain the invariance of rotation, scale change, and brightness, and the computing speed is thrice that of SIFT [38]. The SURF algorithm includes three steps, namely detecting feature points, describing feature points, and matching feature points [39]. The main improvement in SURF when compared to SIFT is in detecting feature points, which is the focus of the introduction of this manuscript.

Hessian matrix is a square matrix that is composed of a second partial derivative of a multivariate function, which describes the local curvature of the function. Additionally, SURF uses a Hessian matrix determinant to approximate the image with the aim to generate mutation points of the image, which constitutes the basis of feature extraction. We assume that the pixel value of the two-dimensional image constitutes a function f(x, y), where (x, y) corresponds to the coordinate of the pixel. A Hessian matrix is obtained for each pixel, as Equation (3) [38]:

(3) $$H\left(f\left(x,y\right)\right)=\left[\begin{array}{cc} \frac{\partial^{2}f}{\partial x^{2}} & \frac{\partial^{2}f}{\partial x\partial y}\\ \frac{\partial^{2}f}{\partial x\partial y} & \frac{\partial^{2}f}{\partial y^{2}} \end{array}\right]$$

The discriminant of the Hessian matrix is given as:

(4) $$\det(H)=\frac{\partial^{2}}{\partial x^{2}}\frac{\partial^{2}f}{\partial y^{2}}-{\left(\frac{\partial^{2}f}{\partial x\partial y}\right)}^{2}$$

When the discriminant of the Hessian matrix reaches the local maximum, it determines that the current point is brighter or darker than other points in the surrounding neighborhood to locate the location of key points. In order to improve the operation speed, SURF uses the box filter to approximately replace the Gaussian filter and improves the calculating speed. The box filter transforms the filtering of the image into addition and subtraction of the sum of the pixels in different regions of the image. It simply searches the integral graph a few times. To balance the error caused by the approximation of the box filter, the approximate value of Hessian matrix determinant of each pixel is considered as Equation (5) [38]:

(5) $$\det(H)=Dxx*Dyy-{\left(0.9*Dxy\right)}^{2}$$

where Dxx and Dyy denote the second-order partial derivatives of function f with respect to x and y axis, respectively, and Dxy denotes the second-order mixed partial derivatives of function f with respect to the x and y axis. It should be noted that the SURF method is similar to the SIFT algorithm in that it uses a three-dimensional linear interpolation method to obtain sub-pixel level displacement.

## 5. Outlier Rejection

**Figure 4:**
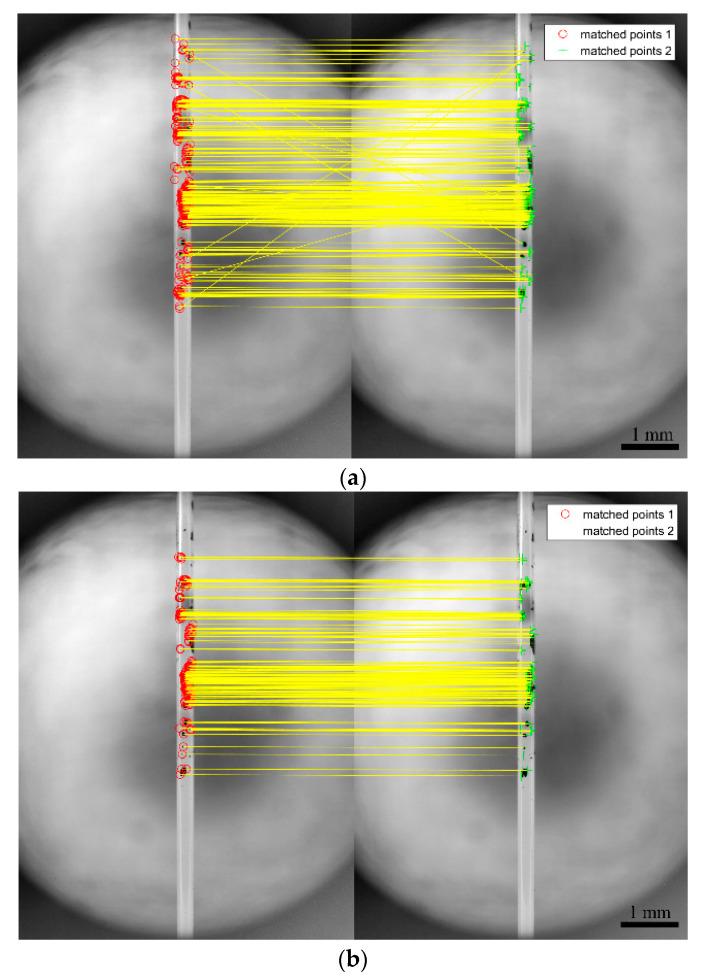
Matched points obtained using (**a**) speeded-up robust feature (SURF) only, and (**b**) SURF and M-estimator sample consensus (MSAC).

Although the SURF algorithm exhibits a series of advantages in feature matching, it is unable to completely avoid generating outliers (i.e., incorrect matching). Therefore, it is necessary to reject the outliers to obtain more accurate and stable strain values. The main methods used to reject outliers from matching features include random sample consensus (RANSAC) [40], progressive sample consensus (PROSAC) [41], and M-estimator sample consensus (MSAC) [42]. The idea of the MSAC algorithm is essentially identical to the RANSAC, which involves randomly selecting a slice of the matching pairs in the rough results, and then using other algorithms (such as affine transformation or projective transformation) to reverse the model and calculate the cost of the model for all rough matching. After repeated iterations, an optimized model is finally obtained to reject all matching pairs that do not conform to the model. In the selection of the noise threshold, RANSAC is more sensitive, and MSAC can partially compensate for some undesirable effects, and thus, it is preferable. Therefore, the study selects projective transformation and the MSAC algorithm to reject outliers. The results before and after the rejected outliers are shown in Figure 4, where Figure 4a denotes the feature points matched by the SURF algorithm, and Figure 4b shows the result after the rejected outliers.

As shown in Figure 4a, there are some outliers in matching feature points, as obtained by the SURF algorithm. Given that the final displacement of feature points corresponds to the average value of the displacement of all feature points, the displacement of these outliers leads to some errors in the final results. Figure 4b shows the matching effect diagram after rejecting the outliers. As shown in the figure, the outliers are also completely removed although the matching feature points are reduced. At the time, the calculated average value corresponds to the real one, and the error is eliminated

## 6. Static Image Analysis

**Figure 5:**
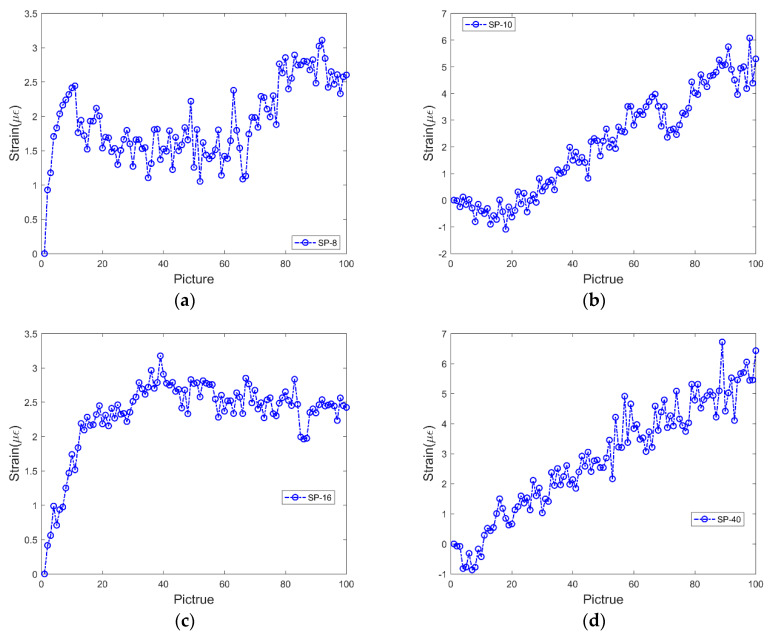
Strain data obtained using smartphones with a resolution of (**a**) 8, (**b**) 10, (**c**) 16, and (**d**) 40 MP.

Although the SURF algorithm has good invariance to brightness and image noise, the smartphone will inevitably be affected by its own and external environment during the shooting process, resulting in the instability of the image. Therefore, it is necessary to measure the displacement of static feature points captured by smartphones. In order to observe the strain of the proposed method under the effect of the external environment and smartphone prior to the experiment, the smartphones with different pixel values are used to capture static feature points to obtain the strain of the feature points in a static image, as shown in Figure 5.

**Figure 6:**
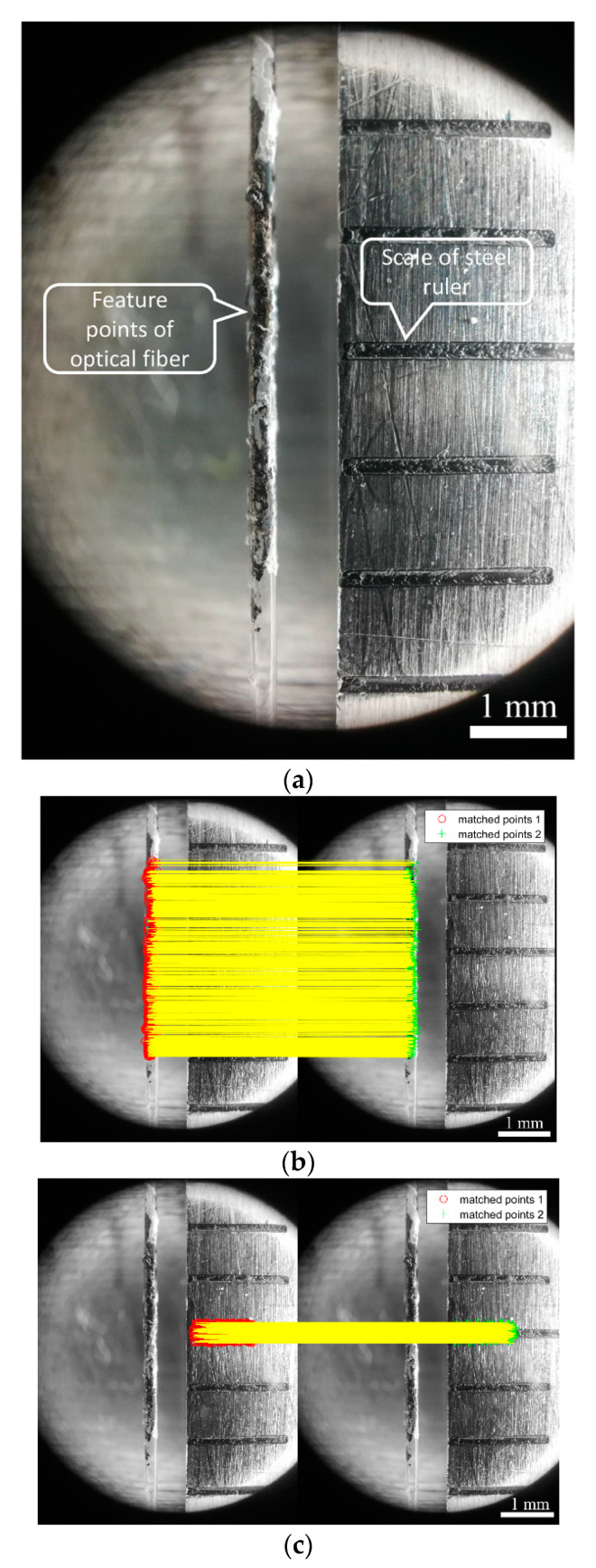
Schematic illustration of the (**a**) Internal layout of micro-scale pictures; (**b**) matched points of the optical fiber; and (**c**) matched points of the steel ruler.

Figure 5 shows the strain value of the feature points obtained by taking 100 pictures continuously by smartphones with different pixel values. As shown in the figure, the strain measured by smartphones with different pixel values increases with an increase in the number of images. Among them, given that 8 and 16 MP pictures were taken by the same smartphone, the strain in the two pictures shows the same growth trend. Similarly, the growth trends of strain obtained by pictures with 10 and 40 MP resolution is also similar. This is mainly because the smartphone’s camera includes a tiny driving lens action for auto focusing, and this results in the focus to drift away from the original position. The type of drift differs with different smartphones, and thus the results of two smartphones are identical. In order to solve the problem, the study proposes a new tracking method, namely the two-marker tracking method, based on the original measuring principle. The measuring principle is that a steel ruler with a notch is placed beside the optical fiber mark point to ensure that the steel ruler exhibits a certain distance from the optical fiber and is in the field of vision of the mobile phone. Simultaneously, the steel ruler should be maintained in the same plane as the optical fiber, as shown in Figure 6a. Second, we trace the markers on the optical fiber and scale on the steel ruler (the light and dark changes near the scale are more prominent and easier to trace), as shown in Figure 6b,c, respectively. Finally, given that the steel ruler is stationary, the displacement of the feature points of the steel ruler corresponds to the displacement of the focus translation of the smartphones. The real displacement of the marker on the optical fiber can be obtained by subtracting the two displacements.

**Figure 7:**
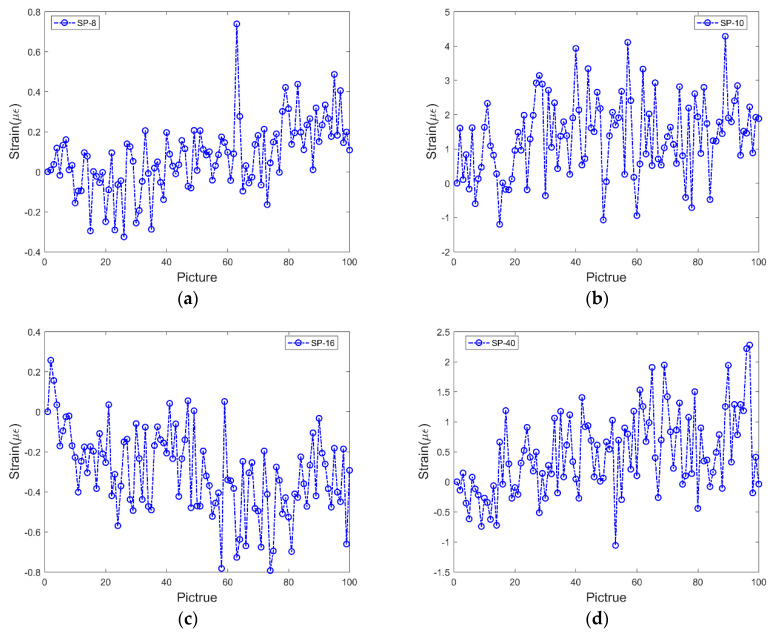
Strain data obtained using the two-marker point tracking method and smartphones with a resolution of (**a**) 8, (**b**) 10, (**c**) 16, and (**d**) 40 MP.

The two-marker point tracking method is applied to recalculate the photographs used in Figure 5, and the new noise is obtained, as shown in Figure 7. As shown in the figure, the result significantly improves after the operation of the new method. The noise values of the four types of pixel images essentially move up and down around the zero axes. This indicates that the new method exhibits lower error and improved stability.

## 7. Analysis of Test Results

**Figure 8:**
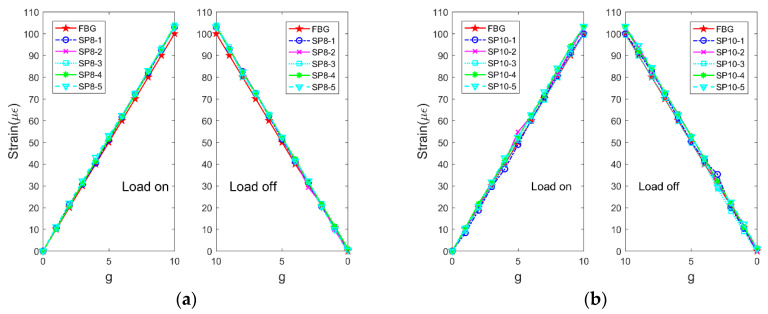

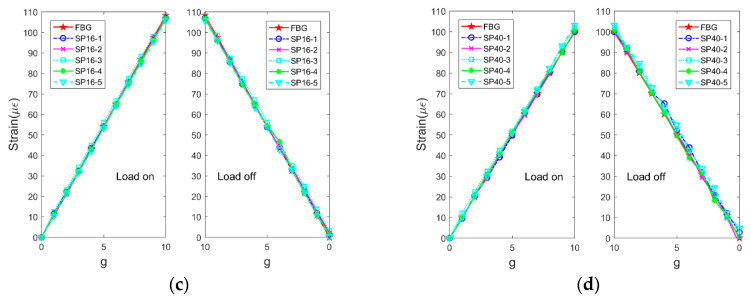
Strain data as measured by fiber Bragg grating (FBG) and smartphones with (**a**) 8, (**b**) 10, (**c**) 16, and (**d**) 40 MP.

In order to observe the accuracy of strain measurement using a smartphone with a microscope, 1 g weights were placed and removed in the pallet connected to the optical fiber to simulate the loading and unloading process. Prior to the experiment, a mass corresponding to 100 g was placed in the pallet in advance to fabricate the optical fiber in the pre-loading state. Specifically, 10 weights were loaded and unloaded at 10 levels. When loading and unloading the weights, the pictures were taken along with the deformation pictures, and the FBG reading was simultaneously recorded for comparative analysis. In order to observe the stability of the method, the above process was repeated five times in each test case. The results are shown in Figure 8.

As shown in the figure, the strain measured using the smartphone with a microscope exhibits good consistency with the strain measured by FBG. However, the actual strain represented by each pixel of smartphones with different pixels is different, and increases in the pixel values increase the accuracy of the smartphone. The accuracy of strain, as measured by smartphones with different pixels, did not significantly change. This was mainly because the SURF method corresponded to a sub-pixel level detection method. The calculated displacement of feature points can be accurate up to four decimal places, which improves the accuracy of strain as measured by the low-pixel smartphone. Additionally, the results of five repeated experiments also matched well, and this indicated that the strain measurement method based on the smart phone exhibited good stability.

**Figure 9:**
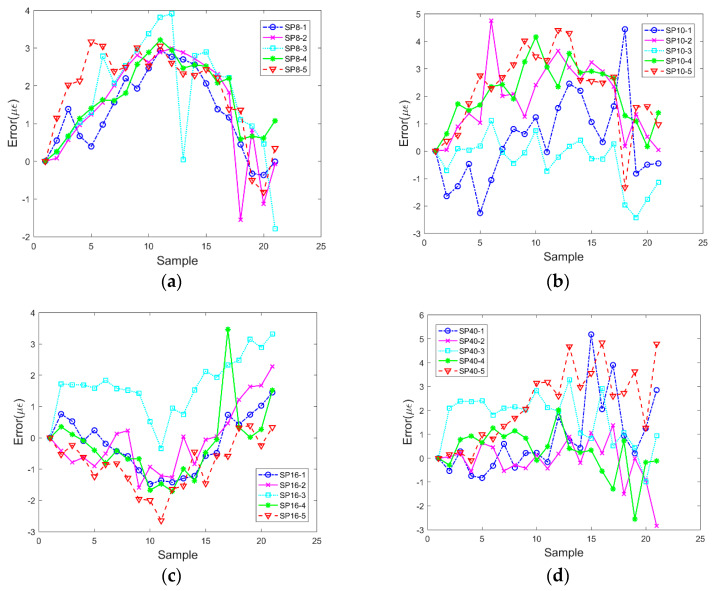
Errors obtained using the data as measured by fiber Bragg grating (FBG) and smartphones with (**a**) 8, (**b**) 10, (**c**) 16, and (**d**) 40 MP.

Based on the data in Figure 8, the errors between the strain data measured by smartphones with different pixels and FBG are calculated, as shown in Figure 9.

As shown in Figure 9, the errors between the two methods are relatively small, and the errors when each test case is repeated five times are relatively discrete. This was mainly due to two factors. The first factor corresponded to the friction between the smartphone and optical fiber during the test and the other factor corresponded to the uncertainty of the displacement detected at the sub-pixel level because placing and removing weights caused a small disturbance to the optical fiber in the process of loading and unloading. The magnitude of the disturbance was random, and the time when the pictures were taken was also random. This caused displacement of feature points in the pictures obtained in different experiments to exhibit certain errors. In order to further verify the accuracy and stability of strain measurement using the smartphone with a microscope, Table 3 lists the maximum and standard error values of the test data (including the results of one-marker point tracking and two-marker point tracking) of four different pixels of smartphones and FBG data.

As shown in Table 3, the maximum error between the strain measured by the two-marker tracking method based on the smartphone and the strain measured by FBG corresponded to 5.2 με, while the maximum value of the standard error corresponded to 2.5 με, which fully satisfies civil engineering requirements. When compared with the one-marker tracking method, the errors obtained by the two-marker point tracking method are generally lower, which shows that the method improved the accuracy of the strain measurement.

## 8. Conclusions

In the study, the accuracy and stability of the proposed method based on SURF to track the displacement of feature points in microimages to measure the strain were verified via experiments. The method can be widely used to obtain microimages by using smartphones and portable mobile phone microscopes. The accuracy of the SURF algorithm matching points and noise obtained by the method under the effect of the external environment were analyzed. Specifically, 1 g weights were used to stretch the optical fiber, and the strain of the optical fiber was measured via the smartphone and FBG. Five repeated tests were performed under each test case, and the results were compared with the FBG measurement. This verified the accuracy and stability of the proposed method. The results are as follows:

(1) The accuracy of using SURF to match feature points on the optical fiber before and after the deformation was analyzed. The results indicated that there were some outliers by simply using the SURF method. The outliers were rejected using the MSAC algorithm to improve the accuracy of the proposed method.
(2) The noise obtained by the proposed method under the effect of the external environment were analyzed. The results indicated that the strain increases with increases in the number of images if only the marker on the optical fiber was tracked. This was because the focus shifted when the smartphone continuously shot using automatic focus. In order to solve the problem, the original method was improved. A fixed steel ruler was added to a side of the marker on the optical fiber, and two markers were simultaneously tracked (which is termed as the two-marker tracking method). The effect of mobile focus translation on the measurement results was eliminated using the new method.
(3) The strain data obtained by the FBG measurement and smartphones using the new method were compared and analyzed. The results indicated that the strains obtained from five repeated experiments with different pixels of the smartphone were in good agreement with the FBG data. The results of analyzing the error of the two methods revealed that the maximum error corresponded to 5.2 με and the maximum standard error corresponded to 2.5 με, which satisfied civil engineering requirements.

In summary, the study improves the accuracy and stability of the method for strain measurement (which used the SURF method) to trace the displacement of feature points in microimages to obtain the strain of an object. It lays the foundation for the development of practical sensors by using smartphones, which also forms the focus of a future study.

## Figures and Tables

**Table 1:** Test cases used in the study.

Test Case	Pixel Value (MP)	Measuring Distance L (cm)	Number of Experiments
1	8	30	5
2	10	30	5
3	16	30	5
4	40	30	5

**Table 2:** Pixel values per mm and actual strain value represented by each pixel for different smartphones.

Pixel Value (MP)	Pixel Value per mm (Pixel)	Measuring Distance L (cm)	με/Pixel
8	505	30	6.6
10	588	30	5.7
16	705	30	4.7
40	1178	30	2.8

**Table 3:** Errors of measurement results using FBG and smartphones.

Pixel Value (MP)	Experiment No.	Weight (g)	One-Marker Tracking Error (με)		Two-Marker Tracking Error (με)	
			Variance	Maximum (Absolute)	Variance	Maximum (Absolute)
8	1	1	1.3	3.3	1.2	2.9
8	2	1	2.3	3.6	1.9	3.0
8	3	1	2.4	4.3	2.2	3.9
8	4	1	2.3	5.2	0.9	3.2
8	5	1	2.2	4.3	1.4	3.2
10	1	1	1.4	4.9	2.4	4.4
10	2	1	1.5	5.4	1.8	4.8
10	3	1	0.9	2.5	0.8	2.4
10	4	1	2.1	4.7	1.2	4.2
10	5	1	2.6	4.7	2.2	4.4
16	1	1	0.7	1.4	0.8	1.4
16	2	1	1.1	3.5	1.1	2.3
16	3	1	0.7	2.6	0.9	3.3
16	4	1	1.3	3.5	1.3	3.5
16	5	1	0.6	2.2	0.7	2.6
40	1	1	6.7	6.6	2.4	5.2
40	2	1	1.0	3.0	0.8	2.8
40	3	1	1.0	3.4	1.1	3.3
40	4	1	1.2	3.2	0.9	2.6
40	5	1	3.0	5.7	2.5	4.8
